# Progress in Surgery

**Published:** 1897-12-25

**Authors:** 


### PROGRESS IN SURGERY.

### GENERAL SURGERY.

(*Continued from page* 206.)

**Fractures and Dislocations.**—The surgical treatment of fractures in former times compared with their treatment to-day formed the subject of a paper by Dr. O. H. Allis[14] before the Philadelphia Academy of Surgery. He especially dwelt upon the open operative measures in so-called simple and compound fractures. While deprecating the resort to these methods in all simple fractures, he urged that very many so-called simple instances of this kind are as grave as the more dreaded compound fractures, and cannot possibly be scientifically treated by any than open measures. He had seen six fractures of the elbow-joint, all simple, that resulted in total loss of function of the forearm and hand, and one of these occurred in the hands of a foremost hospital surgeon. In none of the cases was there any sufficient evidence of the gravity of the original injury to create a suspicion of so serious a result. He had within two years two cases of non-union of fracture—the one a femur, the other a humerus. On resorting to open measures he found the cause of non-union to be muscle interposed between the fragments, a condition that could not possibly be detected by palpation and manipulation at the time of setting the limbs, though both were set under an anæsthetic. He therefore insists that there is nothing simple about a fracture from a surgical standpoint, and as a definition it is both misleading and impolitic, and should be abandoned. The terms open and closed have been advocated; they are easily spoken, easily remembered, familiar, and not misleading. To his mind better terms, though less familiar, are infected and non-infected. Mr. John Poland[15] publishes a paper on the diagnosis of traumatic separation of the epiphysis. He treats of the leading symptoms of these injuries in general, describes the principal features of certain separations, and gives a drawing of a specimen which he had dissected. Practical details as to the employment of the Röntgen process in detecting fractures and dislocations, and a full description of the apparatus to be employed in clinical and other work—as well as the literature of the subject—is to be found in Büttner and Müller's[16] recent work, "Technik und Verwerthung der Röntgen'schen Strahlen." The Röntgen rays in surgery is also the subject of a practical article by Dr. Carl Beck.[17] In taking pictures of the extremities, the position, he says, deserves attention. The forearm is best photographed in supination, although this position is by no means the most comfortable one for the patient. The upper arm and the thigh can be taken in any position. The leg should be photographed while its external surface rests on its support, the knee being bent and the thigh rotated. The foot is best photographed in the direction of the dorsum towards the sole from the toes up to the upper third of the metatarsus. Further backwards the first and third cuneiform bones and scaphoid present an obstacle, so that it is advisable to illuminate the foot on these portions transversely, by having the outer surface rest on the support. In this way the isolated shadows of the astragalus, os calcis, cuboid, scaphoid, and fourth and fifth metatarsal bones can be seen. The hand is taken from the dorsum to the palm. The knee-joint rests best on the external condyle. The humero-ulnar joint is preferably taken transversely, while the humero-radial joint should be illuminated from the flexor to the extensor side. Beck adds that with the fluoroscope the motions of the bones at their joints, the larynx, the hyoid bone, &c., can be studied. Many more points on the same subject are described in Walsh's recent work,[18] "Röntgen Rays in Medical Work," which should be in the hands of every practitioner. The well-known fact that the ingestion of thyroid gland or extract exerts a very favourable action on the development of osseous tissue, as shown by the rapid growth of children with myxœdematous idiocy treated in this way, has suggested to Dr. G. Gauthier[19] that thyroidin might be useful in fractures with retarded consolidation. He has met with two cases, and although these were the only ones in which he had occasion to try this treatment, the results, as given in the *Lyon Médical* for July 11th, were sufficiently good to encourage practitioners in trying thyrotherapy when a fracture fails to unite. Lauderer[20] has lately described as follows the advantages of celluloid as a splint material: A solution of the thickness of syrup is made by immersing scrap celluloid, which is cheap, in acetone, and agitating from time to time, several days being consumed in the process. A plaster of Paris cast of the part to which the corset or splint is to be applied is wound with strips of thin muslin, and over this the solution is applied and rubbed in; then another layer of muslin is wound on and more solution applied, and so on until there are six or eight alternate coats of muslin and celluloid. In a few hours the coating feels firm, and on the following day it may readily be removed from the cast. The splint presents a neat appearance, and is extremely light, weighing not more than half as much as one of water-glass, and not more than a quarter as much as one of plaster of Paris. It has also great firmness, so that for ambulant patients it does not require to be strengthened with straps. On account of its elasticity, it is easy to put on and take off. Its durability is almost unlimited. Moisture, profuse perspiration, or suppuration does not affect it in the least. But its greatest advantage is its simplicity; it can be made by any practitioner. It is easily bent, cut, padded, and trimmed, and may be fortified by using more of the solution. If it does not fit well in any particular place, it can be altered by manipulation, after slightly warming to render it flexible. As to the danger from its ignition, that is not great. A case of gun-shot fracture of the humerus is described by Mr. John M'Ardle,[21] and a radiagraph of the upper extremity gives a clear representation of the situation and extent of the fracture after consolidation. A very unusual instance of fracture of a vertebral articular process in the lumbar region is published by Dr. J. K. Mitchell,[22] in a woman aged 32, who in moving backwards stepped with her foot into a hole and wrenched her spine in the effort to recover her balance. After five years of suffering from paroxysms of pain, accompanied by twitchings and involuntary movements of the leg, an exploratory operation was performed, and a portion of the intravertebral foramen formed by the fourth lumbar vertebra was found to be broken off and displaced forwards. It was, therefore, removed. The patient was relieved at once and a complete recovery followed.

[14] Annals of Surgery, June, 1897, p. 698. [15] Pediatrics, July 14, 1897. [16] Encyklopädie der Photographie, Heft. 28, April 8, 1897. [17] International Medical Magazine, May, 1897 p. 241. [18] Röntgen Rays in Medical Work, Baillière, Tindall, and Cox, 1897. [19] Medical Week, July 16, 1897, and N.Y. Med. Jour., Aug. 14, 1897, p. 235. [20] Wiener Med. Blätter, July 15, 1896; and N.Y. Med. Jour., Sept. 4, 1897, p. 238. [21] Dublin Journ. Med. Science, Sept. 1897, p. 198. [22] University Medical Mag., Philadelphia, June, 1897; and Lancet, July 17, 1897.

